# Genetic predisposition, Aβ misfolding in blood plasma, and Alzheimer’s disease

**DOI:** 10.1038/s41398-021-01380-0

**Published:** 2021-05-01

**Authors:** Hannah Stocker, Andreas Nabers, Laura Perna, Tobias Möllers, Dan Rujescu, Annette M. Hartmann, Bernd Holleczek, Ben Schöttker, Julia Stockmann, Klaus Gerwert, Hermann Brenner

**Affiliations:** 1 Network Aging Research, Heidelberg University, Heidelberg, Germany; 2 Division of Clinical Epidemiology and Aging Research, German Cancer Research Center, Heidelberg, Germany; 3 Medical Faculty, Heidelberg University, Heidelberg, Germany; 4 Department of Biophysics, Competence Center for Biospectroscopy, Ruhr-University Bochum, Bochum, Germany; 5 Faculty of Biology and Biotechnology, Department of Biophysics, Ruhr University Bochum, Bochum, Germany; 6 Department of Translational Research in Psychiatry, Max Planck Institute of Psychiatry, Munich, Germany; 7 Department of Psychiatry, Psychotherapy and Psychosomatics, University of Halle, Halle, Germany; 8 Saarland Cancer Registry, Saarbrücken, Germany

**Keywords:** Biomarkers, Diseases

## Abstract

Alzheimer’s disease is highly heritable and characterized by amyloid plaques and tau tangles in the brain. The aim of this study was to investigate the association between genetic predisposition, Aβ misfolding in blood plasma, a unique marker of Alzheimer associated neuropathological changes, and Alzheimer’s disease occurrence within 14 years. Within a German community-based cohort, two polygenic risk scores (clinical Alzheimer’s disease and Aβ_42_ based) were calculated, *APOE* genotype was determined, and Aβ misfolding in blood plasma was measured by immuno-infrared sensor in 59 participants diagnosed with Alzheimer’s disease during 14 years of follow-up and 581 participants without dementia diagnosis. Associations between each genetic marker and Aβ misfolding were assessed through logistic regression and the ability of each genetic marker and Aβ misfolding to predict Alzheimer’s disease was determined. The Alzheimer’s disease polygenic risk score and *APOE* ε4 presence were associated to Aβ misfolding (odds ratio, 95% confidence interval: per standard deviation increase of score: 1.25, 1.03–1.51; *APOE* ε4 presence: 1.61, 1.04–2.49). No association was evident for the Aβ polygenic risk score. All genetic markers were predictive of Alzheimer’s disease diagnosis albeit much less so than Aβ misfolding (areas under the curve: Aβ polygenic risk score: 0.55; AD polygenic risk score: 0.59; *APOE* ε4: 0.63; Aβ misfolding: 0.84). Clinical Alzheimer’s genetic risk was associated to early pathological changes (Aβ misfolding) measured in blood, however, predicted Alzheimer’s disease less accurately than Aβ misfolding itself. Genetic predisposition may provide information regarding disease initiation, while Aβ misfolding could be important in clinical risk prediction.

## Background

Alzheimer’s disease (AD) is a heritable neurodegenerative disease with pathological changes possible 15–20 years before symptoms^1,2^. The disease is characterized by amyloid plaques and tau tangles in the brain, which can be confirmed in vivo through biomarkers or definitively through postmortem examination^3^.

Cerebrospinal fluid (CSF)-derived or positron emission tomography (PET) imaging biomarkers have been widely established to detect neuropathological changes associated with AD even years before clinical symptoms are present^4^. Recently, amyloid beta (Aβ) has also been measured in blood, as a cost-effective and minimally invasive AD marker^5^.

The heritability of AD has been estimated as high as 79%^2^. Other than *APOE* ε4 (*APOE4*), many common variants with low effect sizes have been confirmed to play a role in AD genetic risk^6^. In very large genome-wide association meta-analyses, more than 20 risk loci have been confirmed^6^. Polygenic risk scores (PRS), which summarize this risk through the summation of risk variants weighted by effects, have been developed to characterize AD genetic risk^7^. AD PRSs have exhibited significant predictive ability of AD diagnosis^7^, however, significant associations with CSF or PET measured Aβ have been less consistent^8–11^. Although several genome-wide association studies have identified associated loci with abnormal Aβ levels measured in CSF or by PET imaging^12–16^, Aβ PRSs that may characterize Aβ specific risk have not been explored. Additionally, the relationship between AD/Aβ genetic risk, as distinguished through a PRS, and Aβ measured in blood has yet to be investigated.

The measurement of Aβ misfolding in blood is one strategy to identify early pathological changes associated to AD. In early stages of amyloid accumulation Aβ experiences a structural change from monomeric, alpha-helical or disordered conformations to β‐sheet‐enriched isoforms, the basis of plaque formation in the brain^17–19^.

The aim of this study was to investigate the association between various genetic predictors (*APOE*, AD PRS, and Aβ PRS), Aβ misfolding in blood plasma, a unique marker of Alzheimer associated neuropathological changes, and Alzheimer’s disease occurrence within 14 years. Additionally, the ability of the genetic risk markers and Aβ misfolding to predict vascular dementia (VD) diagnoses within 14 years was assessed.

## Materials and methods

### Study design and participants

The AD PRS was derived from results of stage 1 of the IGAP meta-analysis^6^, while the Aβ PRS was derived from a genome-wide association study (GWAS)^12^. Both PRSs were applied in a subsample within the prospective community-based cohort, the ESTHER study.

Summary statistics from stage 1 of the IGAP meta-analyses from Kunkle et al.^6^ were utilized, in which genotyped and imputed data on 11,480,632 SNPs was used to meta-analyze four previously-published GWAS consortia datasets consisting of 21,982 AD cases and 41,944 controls^6^.

The Aβ PRS was drawn from summary statistics of the Aβ GWAS by Deming et al. A GWAS of Aβ_42_, tau, and phosphorylated tau levels in CSF from 3146 participants across nine studies was completed to identify novel biological AD associated variants^12^. The associations between 7,358,575 SNPs and low Aβ_42_ measured in CSF were assessed.

The subjects for the analyses for this study were drawn from the ESTHER study, a large community-based cohort study conducted in Saarland, Germany^20,21^. A total of 9940 participants aged 50–75 years were recruited by their general practitioners (GPs) during a general health examination in a statewide study in Saarland, Germany in 2000–2002. Participants completed standardized self‐administered health questionnaires and provided blood samples. Information regarding age, sex, education, medical history, and lifestyle factors was collected at baseline through participant questionnaires and medical records. Follow‐up questionnaires, medical records, and biological samples were collected after 2, 5, 8, 11, 14, and 17 years. The ESTHER study was approved by the Ethics Committee of the Medical Faculty of Heidelberg University and of the Physicians’ Board of Saarland, and all participants gave written informed consent.

**Fig. 1:**
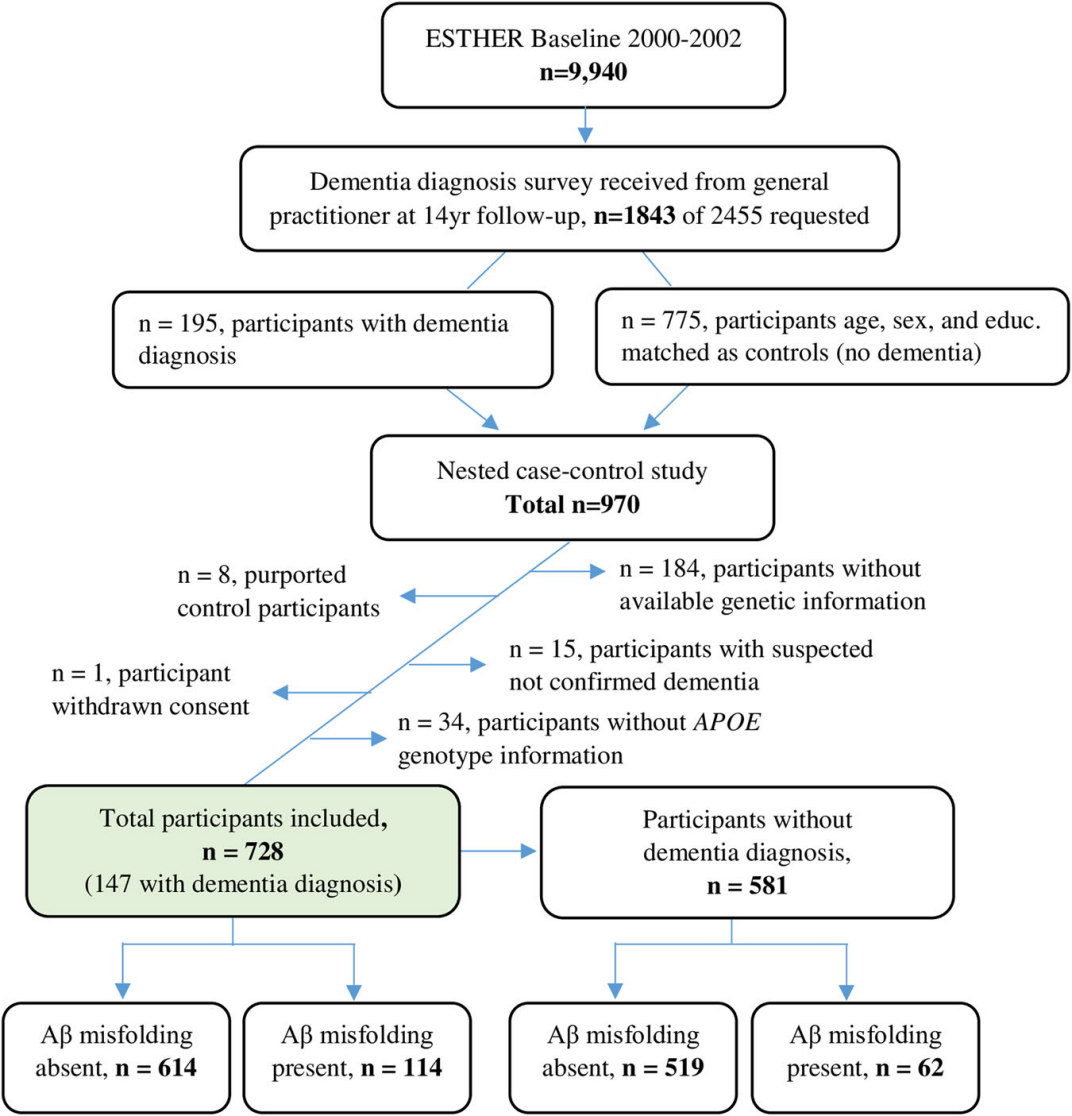
Flow chart of ESTHER study participants included in analyses.

The subsample used for these analyses was a nested case-control study including 970 participants within the ESTHER study^18^. GPs reported patient dementia diagnoses and provided all available medical records from other specialized providers. The current guidelines in Germany for AD diagnosis follow the National Institute on Aging and the Alzheimer’s Association^22^ or the International Working group (IWG)-2 criteria^23,24^, for VD diagnosis the National Institute of Neurological Disorders and Stroke (NINDS)-Association Internationale pour la Recherche et l’Enseignement en Neurosciences (AIREN) criteria^25^. Excluded participants in this study included 184 participants without available genotyped or imputed genetic data for the PRSs, 15 cases where suspected dementia diagnosis could not be confirmed by further medical records, seven purported controls with a later identified dementia diagnosis, 34 participants without *APOE* genotype data, and one participant that withdrew informed consent (Fig. 1).

### Genotyping

Blood samples taken at baseline and stored at −80 °C were used for genetic analyses. DNA from whole blood samples was extracted using a salting out procedure. *APOE* data was directly genotyped and determined based on allelic combinations of the SNPs rs7412 and rs429358 using predesigned TaqMan SNP genotyping assays (Applied Biosystems, Foster City, CA). Genotypes were analyzed in an endpoint allelic discrimination read using the Bio‐RAD CFX Connect System (Bio‐Rad Laboratories, Hercules, CA).

Later, genome-wide SNP analyses were performed from extracted whole blood DNA using the Illumina Infinium OncoArray and Global Screening Array BeadChips (Illumina, San Diego, CA, USA)^26^. General genotyping quality control assessment was done following the Nature Protocols article from Anderson et al.^27^. Imputation of the quality controlled data was conducted using the Michigan Imputation Server, where SHAPEIT2 was used to phase the data and Minimac 4 was used to impute to the HRC Version r1.1 ^24^ reference panel^28,29^.

### Aβ misfolding measurement

The blood plasma samples used in this study were collected at baseline and used to measure Aβ misfolding in each participant. The Aβ misfolding marker assessed in these analyses detects the change in the Aβ peptides secondary structure through an immuno-infrared-sensor (WO 2015121339 A1), the details of which have been reported elsewhere^18,30,31^. This structure-based biomarker examines the misfolding state of Aβ in blood plasma. In agreement with a previously validated spectral threshold^18^, participants with a cutoff of < 1642 cm^−1^ were considered Aβ misfolding positive or to have high misfolding. Those participants at or above the validated spectral threshold were considered Aβ misfolding negative or to have low misfolding. The validated spectral threshold portrays the point at which the maximum position of the amide I absorbance band indicates an abnormal Aβ state associated to AD pathology. A plot of the distribution of Aβ misfolding can be found in Supplementary Fig. 1. At this threshold, we have previously shown 71 and 69% sensitivity and 91 and 86% specificity to detect AD cases in the ESTHER and BioFINDER studies, respectively^18^. Additionally, Aβ misfolding has shown significant correlations to Aβ burden measured by PET scan and in CSF^18^. The immuno-infrared-sensor has been validated in detail, including generation and characterization of NHS-silane, antibody batch-to-batch variation, antibody performance with synthetic and standard reference CSF and blood plasma samples, matrix effects, lower and higher limits of quantification, assay selectivity, sample handling, and documentation of zero background signals after Aβ immunodepletion^17,18,30–32^. The Aβ secondary structure distribution is detected as a relative measure and is independent of concentration fluctuations and sample variation. All plasma sample analyses were performed in a blinded manner at the department of Biophysics at Bochum University, Germany.

### Polygenic risk score calculation

The AD PRS and Aβ PRS in this study were weighted scores including AD and Aβ associated SNPs, calculated by summing the number of risk alleles weighted by the magnitude of association to AD (ln of the odds ratio (OR)) from Kunkle et al.^6^ or Aβ from Deming et al.^12^.

For the AD PRS, SNPs reaching genome-wide significance in the IGAP meta-analysis were extracted from the imputed ESTHER data (*n* = 1234). Linkage disequilibrium-based clumping was carried out, providing the most significantly associated SNP in each region of linkage disequilibrium (using PLINK clumping command with a pairwise *r*^2^ threshold of 0.2) leaving 106 SNPs. Then, SNPs within or directly upstream/downstream from the *APOE* locus (chr19: 45,404,000–45,418,000) were excluded (*n* = 9). Finally, a minor allele frequency (MAF) threshold of 0.01 was applied excluding 25 SNPs. The remaining SNPs (*n* = 72) had imputation quality median *R*^2^ = 0.92 (*R*^2^ range: 0.47–0.99).

The same procedure was used for the Aβ PRS using summary statistics from Deming et al., where 133 SNPs were extracted from the imputed ESTHER data, and 21 SNPs remained after linkage disequilibrium-based clumping, all of which remained after applying the MAF threshold. The SNPs included in the Aβ PRS had imputation quality median *R*^2^ = 0.97 (*R*^2^ range: 0.83–0.99). Neither PRS included SNPs located within the *APOE* locus.

The scores were normalized by subtracting the mean and dividing by the standard deviation (SD), which were both calculated from the overall sample. For the sake of comparability of prediction performance of PRS and *APOE*, the cutoff for PRS+ was determined as the score point in which the number of PRS+ individuals was proportionate to the number of *APOE4*+ (≥ 1 ε4 allele) individuals in the Aβ misfolding negative group. It should be noted that this not a true or validated threshold but was chosen for comparability with *APOE* only. The distribution and cutoff values for each of the PRSs are described in Supplementary Fig. 1. Using the method described above, participants at or above the 73.6 percentile were considered AD PRS+ and those below AD PRS−. For the Aβ PRS, participants at or above the 74.7 percentile were considered Aβ PRS+ and those below Aβ PRS−. The PRSs were additionally investigated as continuous variables (per SD increase) and as categorical variables (quintiles).

### Statistical analyses

Descriptive statistics were calculated to provide information on participant characteristics, while chi-square and *t*-tests were used to compare Aβ misfolding positive and Aβ misfolding negative groups in all participants and participants without dementia diagnosis.

Multivariable logistic regression models were used to assess the associations between the Aβ PRS, AD PRS, *APOE*, and Aβ misfolding, in cross-sectional analyses. Logistic regression was also used to assess the ability of Aβ misfolding (for comparison), both PRSs, and *APOE4* to predict AD and VD diagnoses within 14 years. The PRSs were considered per SD increase in score, as a binary variable following the cutoff previously described, and as quintiles. *APOE* status was utilized as a binary variable (*APOE4*+:≥1 ε4 allele vs. *APOE4*−: no ε4 allele). Additionally, each of the PRSs and *APOE4* status were combined and odds ratios were calculated for individuals that were: (1) both PRS+ and *APOE4*+; (2) PRS− and *APOE4*+; and (3) PRS+ and *APOE4−* all compared to the reference PRS− and *APOE4*−. Both Aβ PRS and AD PRS status were combined in a similar fashion. Covariates for all logistic regression analyses included age, sex, 10 principal components, and education, measured by years of formal education (≤9, 10–11, ≥12 years; standard categories of the German school system; the lowest category corresponds to a leaving certificate from school, the highest category corresponds to qualification for university). ORs including 95% confidence intervals (CIs) were calculated to assess associations with Aβ misfolding in all participants and in those participants without known dementia diagnosis. In the analyses with endpoints AD and VD, all participants were considered. Education data missing at random was imputed three times following the Markov chain Monte Carlo (MCMC) method^33^.

Receiver operating characteristic (ROC) curve analysis was completed for each PRS, *APOE*, and Aβ misfolding, where the PRSs and Aβ misfolding were considered continuously and *APOE* was considered categorically (*APOE* ε2ε2, ε2ε3, ε3ε4, ε4ε4 vs. ε3ε3). For AD diagnosis within 14 years, ROC curves and corresponding area under the curves (AUCs) were calculated based upon: (1) Aβ PRS; (2) AD PRS; (3) *APOE*; and (4) Aβ misfolding. Additionally, for explicit comparison with *APOE*, ROC curves were also calculated for (1) *APOE* + Aβ PRS; (2) *APOE* + AD PRS; (3) *APOE* + Aβ misfolding; and (4) for all predictors together *APOE* + AD PRS + Aβ PRS + Aβ misfolding. ROC contrast analysis using the DeLong test was conducted to compare for significant differences between curves^34^.

Additionally, AUC values were calculated for outcome Aβ misfolding (continuous) by the Aβ PRS (continuous), AD PRS (continuous), and *APOE* (categorical). Spearman rank correlation coefficients were calculated to assess the correlation between each of the genetic predictors (*APOE*: ordinal, i.e., number of *APOE4* alleles, Aβ PRS: continuous, AD PRS: continuous).

All analyses were two-sided, conducted at an α-level 0.05, and completed using SAS software, version 9.4 (SAS Institute, Cary, NC).

## Results

### Participant characteristics

A flow chart of the participants included in this study is expressed in Fig. 1 and detailed participant characteristics are shown in Table 1. The analyses consisted of 728 participants with 114 participants considered Aβ misfolding positive and 614 participants Aβ misfolding negative. Of the 728 total participants, 59 had an AD diagnosis, 54 a VD diagnosis, 34 a mixed dementia diagnosis, and 581 remained without a dementia diagnosis throughout the 14-year follow-up.

**Table 1:** Participant characteristics.

	All		Participants without dementia diagnosis			
	Aβ+	Aβ−	Aβ +	Aβ−	*p*-value^a^	*p*-value^b^
*n*	114	614	62	519		
Age, mean ± SD (range)	68.4 ± 4.8 (54–75)	68.5 ± 4.7 (52–75)	67.9 ± 5.1 (54–75)	68.4 ± 4.7 (53–75)	0.81	0.44
≤70 years, *n* (%)	59 (51.8)	307 (50.0)	36 (58.1)	262 (50.5)	0.73	0.26
>70 years, *n* (%)	55 (48.3)	307 (50.0)	26 (41.9)	257 (49.5)		
Male, *n* (%)	57 (50.0)	251 (40.9)	34 (54.8)	210 (40.5)	0.07	0.03
Female, *n* (%)	57 (50.0)	363 (59.1)	28 (45.2)	309 (59.5)		
≤9 yrs education, *n* (%)	99 (86.8)	533 (88.1)	54 (87.1)	452 (87.8)	0.12	0.45
10–11 yrs education, *n* (%)	5 (4.4)	44 (7.3)	3 (4.8)	38 (7.4)		
≥12 yrs education, *n* (%)	10 (8.8)	28 (4.6)	5 (8.1)	25 (4.8)		
Aβ PRS−, n (%)	83 (72.8)	461 (75.1)	48 (77.4)	389 (75.0)	0.61	0.67
Aβ PRS+, *n* (%)	31 (27.2)	153 (24.9)	14 (22.6)	130 (25.0)		
AD PRS−, *n* (%)	76 (66.7)	460 (74.9)	45 (72.6)	392 (75.5)	0.07	0.61
AD PRS+, *n* (%)	38 (33.3)	154 (25.1)	17 (27.4)	127 (24.5)		
*APOE4*−, *n* (%)	74 (64.9)	460 (74.9)	43 (69.4)	395 (76.1)	0.03	0.24
*APOE4*+, *n* (%)	40 (35.1)	154 (25.1)	19 (30.6)	124 (23.9)		
Aβ PRS− *APOE4*−, *n* (%)	69 (60.5)	408 (66.5)	40 (64.5)	348 (67.0)	0.10	0.40
Aβ PRS+ *APOE4*−, *n* (%)	5 (4.4)	52 (8.5)	3 (4.8)	47 (9.1)		
Aβ PRS− *APOE4*+, *n* (%)	14 (12.3)	53 (8.6)	8 (12.9)	41 (7.9)		
Aβ PRS+ *APOE4*+, *n* (%)	26 (22.8)	101 (16.5)	11 (17.8)	83 (16.0)		
AD PRS− *APOE4*−, *n* (%)	68 (59.7)	413 (67.2)	40 (64.5)	354 (68.2)	0.06	0.57
AD PRS+ *APOE4*−, *n* (%)	6 (5.3)	47 (7.7)	3 (4.8)	41 (7.9)		
AD PRS− *APOE4*+, *n* (%)	8 (7.0)	47 (7.7)	5 (8.1)	38 (7.3)		
AD PRS+ *APOE4*+, *n* (%)	32 (28.1)	107 (17.4)	14 (22.6)	86 (16.6)		
AD PRS− Aβ PRS−, *n* (%)	70 (61.4)	408 (66.5)	42 (67.8)	348 (67.0)	0.25	0.76
AD PRS+ Aβ PRS−, *n* (%)	13 (11.4)	53 (8.6)	6 (9.7)	44 (8.5)		
AD PRS− Aβ PRS+, *n* (%)	6 (5.3)	52 (8.5)	3 (4.8)	41 (7.9)		
AD PRS+ Aβ PRS+, *n* (%)	25 (21.9)	101 (16.5)	11 (17.7)	86 (16.6)		

*p*-values reported are for comparisons between Aβ misfolding positive and negative participants for entire sample^a^ and participants without dementia diagnosis^b^.

*Aβ+* Aβ misfolding positive, *Aβ−* Aβ misfolding negative, *Aβ PRS* Aβ specific polygenic risk score, *AD PRS* Alzheimer’s disease polygenic risk score, *APOE* apolipoprotein E

The mean age of all participants was 68.5 years at ESTHER baseline when the blood samples were taken and used for Aβ measurements. Among Aβ misfolding positive participants, 27% were Aβ PRS+, 33% were AD PRS+, and 35% were *APOE4*+. Among Aβ misfolding negative participants, 25% were Aβ PRS+, 25% were AD PRS+, and 25% were *APOE4*+. This was due to the way in which the PRS cutoffs were selected (PRS positivity was determined as the score point in which the number of PRS+ individuals was proportionate to the number of *APOE4*+ (≥1 ε4 allele) individuals in the Aβ misfolding negative group).

### Association of AD & Aβ genetic risk and Aβ misfolding

#### Among all participants

The AD PRS per SD increase in score and as a binary variable were significantly associated with Aβ misfolding (OR, 95% CI: AD PRS per SD: 1.25, 1.03–1.51; AD PRS+: 1.58, 1.01–2.46) (Table 2). *APOE4* positivity was also significantly associated with Aβ misfolding (OR, 95%CI: 1.61, 1.04–2.49). The results of the PRSs as categorical variables (quintiles) can be found in Supplementary Table 1.

**Table 2:** Logistic regression results: Association between Alzheimer’s and Aβ polygenic risk scores and Aβ misfolding.

	All, *n* = 728				Participants without dementia diagnosis, *n* = 581			
	n, Aβ+	*n*, Aβ−	OR (95% CI)	*p*-value	*n*, Aβ+	*n*, Aβ−	OR (95% CI)	*p*-value
Aβ PRS per SD	114	614	1.05 (0.85-1.29)	0.67	62	519	1.03 (0.77–1.38)	0.85
Aβ PRS per SD*	114	614	0.89 (0.70–1.15)	0.38	62	519	0.95 (0.67-1.32) +4)	0.74
AD PRS per SD	114	614	**1.25 (1.03–1.51)**	**0.03**	62	519	1.04 (0.78–1.38)	0.79
AD PRS per SD*	114	614	1.16 (0.90–1.48)	0.25	62	519	0.95 (0.67–1.34)	0.75
Aβ PRS−	83	461	Ref.		48	389	Ref.	
Aβ PRS+	31	153	1.14 (0.72–1.81)	0.58	14	130	0.85 (0.44–1.64)	0.64
AD PRS−	76	460	Ref.		45	392	Ref.	
AD PRS+	38	154	**1.58 (1.01–2.46)**	**<0.05**	17	127	1.13 (0.61–2.12)	0.70
*APOE4*−	74	460	Ref.		43	395	Ref.	
*APOE4*+	40	154	**1.61 (1.04–2.49)**	**0.03**	19	124	1.33 (0.72–2.46)	0.36
Aβ PRS− *APOE4*−	69	408	Ref.		40	348	Ref.	
Aβ PRS+ *APOE4−*	5	52	0.53 (0.20–1.40)	0.20	3	47	0.51 (0.15–1.76)	0.29
Aβ PRS− *APOE4*+	14	53	1.43 (0.73–2.79)	0.30	8	41	1.46 (0.59–3.61)	0.41
Aβ PRS+ *APOE4*+	26	101	1.57 (0.94–2.62)	0.09	11	83	1.14 (0.54–2.39)	0.74
AD PRS− *APOE4*−	68	413	Ref.		40	354	Ref.	
AD PRS+ *APOE4*−	6	47	0.76 (0.30–1.89)	0.55	3	41	0.54 (0.15–1.96)	0.35
AD PRS− *APOE4*+	8	41	0.88 (0.39–2.00)	0.76	5	38	0.94 (0.33–2.73)	0.91
AD PRS+ *APOE4*+	32	107	**1.92 (1.18–3.12)**	**<0.01**	14	86	1.43 (0.72–2.85)	0.31
AD PRS− Aβ PRS−	70	408	Ref.		42	348	Ref.	
AD PRS+ Aβ PRS−	13	53	1.46 (0.73–2.91)	0.28	6	44	1.05 (0.39–2.81)	0.92
AD PRS− Aβ PRS+	6	52	0.62 (0.25–1.53)	0.30	3	41	0.51 (0.15–1.73)	0.28
AD PRS+ Aβ PRS+	25	101	1.53 (0.91–2.57)	0.11	11	86	1.07 (0.51–2.25)	0.86

Model covariates included age, sex, education and 10 principal components.

Bolded results indicate statistical significance, *p* < 0.05.

*Additionally adjusted for *APOE* status

*Aβ+* Aβ misfolding positive, *Aβ−* Aβ misfolding negative, *APOE4*+, apolipoprotein E ≥ 1 ε4 allele, *PRS* genetic risk score, *Ref.* reference, *SD* standard deviation.

#### Among participants without dementia diagnosis

There were no significant associations between Aβ misfolding and any of the included predictors **(**Table 2**)**.

#### Prediction of AD and VD diagnoses by Aβ and AD genetic risk

**Fig. 2:**
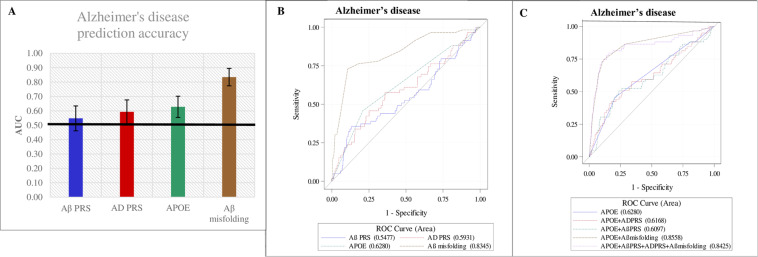
Prediction of AD diagnosis within 14 years by Aβ PRS, AD PRS, *APOE*, and Aβ misfolding: ROC curve analysis. **A** Area under the curve (AUC) values with 95% confidence intervals (CIs); **B** receiver operating characteristic (ROC) curves for individual predictors; **C** ROC curves for comparison of each predictor to *APOE.* AD, Alzheimer’s disease; *APOE*, apolipoprotein E; PRS, polygenic risk score. **B** AUC, 95% CI: Aβ PRS: 0.55, 0.46–0.63; AD PRS: 0.59, 0.51–0.68; *APOE*: 0.63, 0.55–0.70; Aβ misfolding: 0.84, 0.78–0.90C) AUC, 95% CI: *APOE*: 0.63, 0.55–0.70; *APOE* + AD PRS: 0.62, 0.53–0.70; *APOE* + Aβ PRS: 0.61, 0.53–0.69; *APOE* + Aβ misfolding: 0.86, 0.80–0.91; *APOE* + AD PRS + Aβ PRS + Aβ misfolding: 0.84, 0.78–0.91. *P*-values for ROC contrast analysis between *APOE* alone and *APOE* + additional predictor: *APOE* - (*APOE* + AD PRS): *p* = .52; *APOE* - (*APOE* + Aβ PRS): *p* = .26; *APOE* - (*APOE* + Aβ misfolding): *p* < .0001; *APOE*− (*APOE* + AD PRS + Aβ PRS + Aβ misfolding): *p* < 0.0001.

The ability of the Aβ PRS and AD PRS to predict AD diagnosis is shown in Table 3 and Supplementary Table 2. The prediction ability measured by AUC of the Aβ PRS, AD PRS, *APOE*, and Aβ misfolding as well as ROC contrast analyses for comparison of *APOE* to the additional predictors is depicted in Fig. 2.

**Table 3:** Logistic regression results: prediction of AD and VD diagnoses by Alzheimer’s and Aβ polygenic risk.

	AD Diagnosis				VD Diagnosis			
	AD	ND	OR (95% CI)	*p*-value	VD	ND	OR (95% CI)	*p*-value
Aβ PRS per SD	59	581	**1.32 (1.01–1.73)**	**<0.05**	54	581	1.05 (0.78–1.42)	0.76
Aβ PRS per SD*	59	581	1.02 (0.74–1.40)	0.93	54	581	0.98 (0.69–1.41)	0.92
AD PRS per SD	59	581	**1.47 (1.16–1.87)**	**<0.01**	54	581	1.13 (0.85–1.50)	0.41
AD PRS per SD*	59	581	1.21 (0.89–1.66)	0.22	54	581	1.09 (0.75–1.59)	0.64
Aβ PRS−	37	437	Ref.		41	437	Ref.	
Aβ PRS+	22	144	**1.85 (1.05–3.28)**	**0.04**	13	144	0.90 (0.46–1.77)	0.76
AD PRS−	34	437	Ref.		37	437	Ref.	
AD PRS+	25	144	**2.29 (1.30–4.02)**	**<0.01**	17	144	1.34 (0.71–2.50)	0.37
*APOE4*−	32	438	Ref.		37	438	Ref.	
*APOE4*+	27	143	**2.69 (1.54–4.72)**	**<0.001**	17	143	1.26 (0.67–2.37)	0.47
Aβ PRS− *APOE4*−	28	388	Ref.		34	388	Ref.	
Aβ PRS+ *APOE4−*	4	50	1.09 (0.36–3.28)	0.88	3	50	0.60 (0.17–2.14)	0.43
Aβ PRS− *APOE4*+	9	49	**2.61 (1.14–5.98)**	**0.02**	7	49	1.37 (0.54–3.48)	0.51
Aβ PRS+ *APOE4*+	18	94	**2.78 (1.45–5.32)**	**<0.01**	10	94	1.11 (0.52–2.38)	0.79
AD PRS− *APOE4*−	29	394	Ref.		32	394	Ref.	
AD PRS+ *APOE4*−	3	44	0.92 (0.26–3.21)	0.89	5	44	1.58 (0.56–4.47)	0.39
AD PRS− *APOE4*+	5	43	1.66 (0.59–4.65)	0.35	5	43	1.33 (0.47–3.79)	0.59
AD PRS+ *APOE4*+	22	100	**3.09 (1.68–5.70)**	**<0.001**	12	100	1.32 (0.64–2.72)	0.46
AD PRS− Aβ PRS−	29	390	Ref.		32	390	Ref.	
AD PRS+ Aβ PRS−	8	47	2.22 (0.93–5.26)	0.09	9	47	2.36 (1.01–5.52)	<.05
AD PRS− Aβ PRS+	5	47	1.39 (0.50–3.83)	0.61	5	47	1.19 (0.43–3.32)	0.73
AD PRS+ Aβ PRS+	17	97	**2.47 (1.28–4.74)**	**<0.01**	8	97	0.94 (0.41–2.15)	0.87

Model covariates included age, sex, education, and 10 principal components.

Bolded results indicate statistical significance, p < 0 .05.

*Additionally adjusted for *APOE* status.

*AD* Alzheimer’s disease, *APOE4*+ apolipoprotein E ≥ 1 ε4 allele, *ND* participants without dementia diagnosis, *PRS* genetic risk score, *SD* standard deviation, *VD* vascular dementia.

The Aβ PRS was predictive of AD diagnosis per SD increase in score (OR, 95%CI: 1.32, 1.01–1.73) and Aβ PRS+ participants had 85% greater odds of AD diagnosis than Aβ PRS− participants (OR, 95%CI: 1.85, 1.05–3.28). The AD PRS was also predictive of AD diagnosis per SD increase in score (OR, 95%CI: 1.47, 1.16–1.87) and AD PRS+ participants had 2.3 fold odds of AD diagnosis (OR, 95%CI: 2.29, 1.30–4.02). *APOE4*+ participants also had increased odds of AD diagnosis (OR, 95%CI: 2.69, 1.54–4.72).

Aβ misfolding exhibited superior AD diagnosis prediction ability compared to the genetic markers (AUC, 95% CI: Aβ PRS: 0.55, 0.46–0.63 AD PRS: 0.59, 0.51–0.68; *APOE* ε4: 0.63, 0.55–0.70; Aβ misfolding: 0.84, 0.78–0.90) (Fig. 2).

**Fig. 3:**
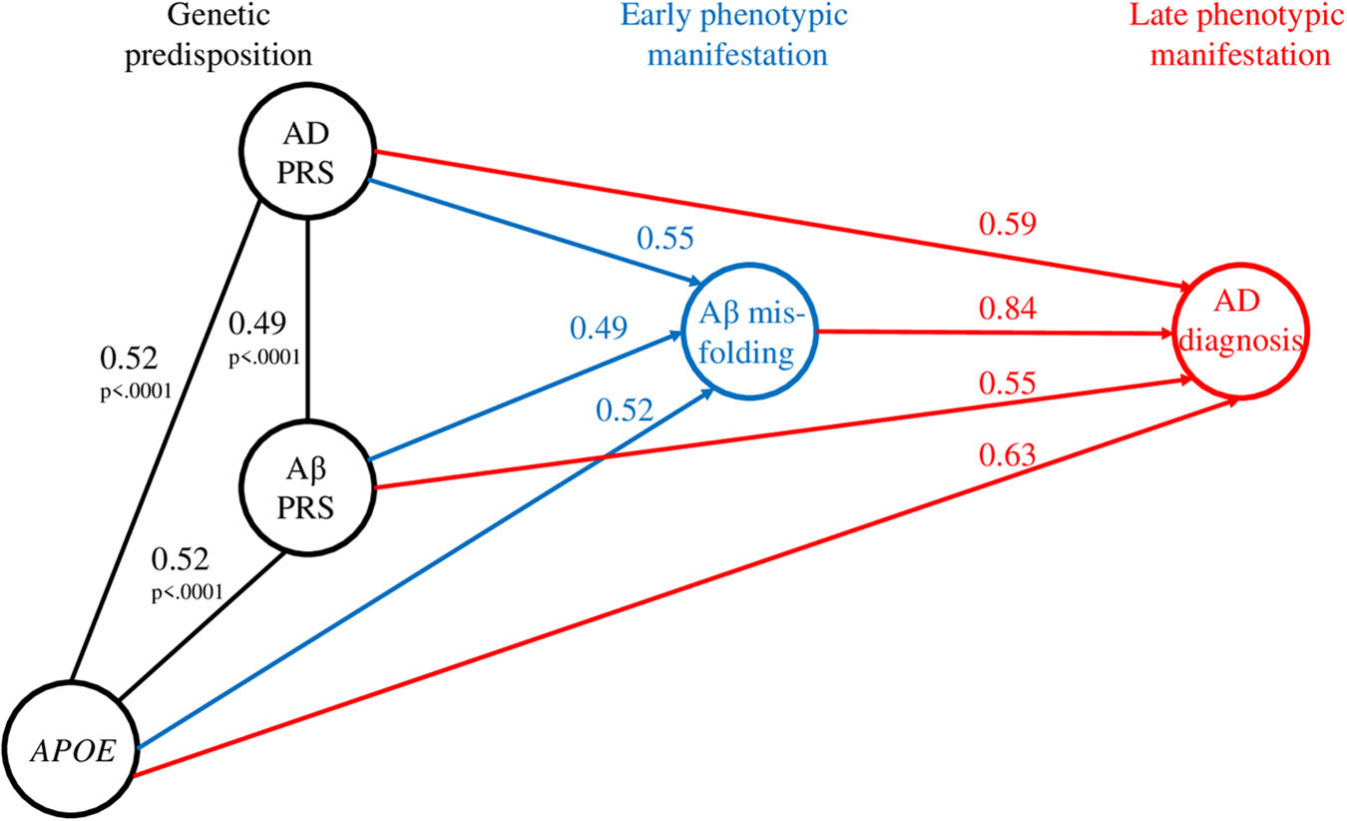
Overarching relationship between the genetic risk markers, Aβ misfolding, and AD diagnosis measured in area under the curve (AUC) values (shown in blue and red) and Spearman correlation coefficients (shown in black). The prediction of Aβ misfolding by the genetic risk markers and AD diagnosis by the genetic risk markers and Aβ misfolding is expressed as AUC values and the relationship between genetic risk markers is expressed as Spearman correlation coefficients.

The relationship between the genetic risk markers, Aβ misfolding, and AD diagnosis is portrayed in Fig. 3 as AUC values and Spearman correlation coefficients. The genetic risk markers were moderately correlated, with correlation coefficients ranging from 0.49 to 0.52. The prediction ability of the genetic risk markers was greater for AD diagnosis than Aβ misfolding status. Among the genetic risk markers, *APOE* predicted both AD and Aβ PRS status. However, the prediction ability of Aβ misfolding for AD exceeded the prediction ability of any of the genetic markers.

The Aβ PRS, AD PRS, and *APOE4* were not significantly predictive of VD diagnosis (Table 3). ROC curve analysis also revealed a lack of or minimal predictive ability of the genetic risk markers and Aβ misfolding to predict VD (AUC, 95% CI: Aβ PRS: 0.52, 0.45–0.61; AD PRS: 0.56, 0.48–0.64; *APOE:* 0.50, 0.42–0.57; Aβ misfolding: 0.54, 0.46–0.63).

## Discussion

This study provides a comprehensive assessment of the interrelationship of various genetic predictors (*APOE*, AD PRS, and Aβ PRS), Aβ misfolding in blood, and AD diagnosis. Aβ misfolding in blood was significantly associated to an AD PRS and *APOE4* status in a community-based cohort of older adults. There was a lack of association between the Aβ PRS and Aβ misfolding; however, the Aβ PRS significantly predicted AD diagnosis within 14 years. Aβ misfolding, a marker of early AD pathological changes, was far more predictive of AD diagnosis than the genetic risk markers.

Although this work is unique in the investigation of AD polygenic risk and Aβ misfolding in blood, previous studies have examined the relationship between an AD PRS and Aβ measured in CSF, by PET imaging, or postmortem. No previous study has utilized an Aβ specific PRS. Previous AD PRSs that included *APOE* exhibited positive associations to CSF Aβ^10,35,36^ or post-mortem measured Aβ^37^. However, in studies utilizing AD PRSs that excluded *APOE*, the association to CSF, PET, and post-mortem measured Aβ was mixed^8,9,11,38–42^, aligning with our results. AD PRSs have shown consistent associations to clinical AD diagnosis^7^, while the same/similar scores were less consistently associated to Aβ^9,40,41^.

Our results lacked significant associations between the Aβ PRS and Aβ misfolding, possibly because the Aβ PRS was based on a GWAS from Deming et al., where SNPs were identified with association to CSF-measured Aβ_42_, whereas Aβ misfolding examines the secondary structure changes of Aβ^17^. Additionally, while the GWA meta-analyses for AD have been large including 94,437 clinical AD cases^6,43^, Aβ specific GWAS have been smaller including at most 3,146 individuals^12,15^.

*APOE* has been more consistently reported to be significantly associated to Aβ^11,35,36,40^. It has been theorized that *APOE* contributes to amyloid accumulation and the AD PRS (additional associated variants) to AD conversion^40^. Additional associated variants included in the PRS were associated to clinical AD after symptom manifestation and, therefore, it may be possible that these variants could be associated to other drivers of AD progression, while *APOE* plays an inherent role in the disease initiation process of AD.

Interestingly, the associations between *APOE*/AD PRS and Aβ misfolding were not evident in participants without dementia diagnoses, possibly because those at high genetic risk of AD experience Aβ misfolding earlier and, therefore, also a diagnosis earlier. Although genetics play an important role in the development of AD, many other factors are involved in the manifestation of clinical AD^44^. AD is inherently very complex; with many medical, lifestyle, and social risk factors that play a role in development.

Aβ misfolding expressed greater AD predictive ability than any of the genetic markers. We have previously shown the odds of AD diagnosis in participants with high Aβ misfolding are 23 times that of participants without Aβ misfolding^19^. Aβ misfolding is a marker of early phenotypic manifestation of AD and can occur many years before clinical symptoms. While genetic risk markers provide important information regarding the risk and pathogenesis of AD, risk prediction by Aβ misfolding is stronger.

### Implications

Most treatments after major Aβ accumulation have failed in the prevention of AD progression even when Aβ itself has been reduced^45^. Therefore, those at highest risk might benefit most from preventative treatment before amyloid accumulation. While there may be little clinical applicability without any available effective prevention or disease-modifying therapies, polygenic risk could play an important role in AD preventative research. Although PRSs can provide risk information at any time throughout life, even early in life before any amyloid accumulation, the predictive values for AD risk are low. It, therefore, appears questionable to what extent testing and preventative measures should be used based on such limited predictive value.

Aβ misfolding was shown to have much stronger predictive value for AD within 14 years before AD diagnosis, in a time frame where Aβ accumulation in the brain may still be limited. Aβ misfolding measurements could be crucial in identifying those individuals who would benefit most from AD symptom preventative measures and disease progression modifying therapeutic treatment. Further research should examine how early in the pathogenic process Aβ misfolding could identify those at highest AD risk and the effectiveness of preventative measures employed at that time point.

### Strengths and limitations

The strengths of this study include the large community-based cohort that has been followed for over 14 years with available genetic, dementia, and Aβ blood plasma information. This study comprehensively assessed genetic predisposition (AD PRS, Aβ PRS, and *APOE*), early pathological changes (Aβ misfolding), and late phenotypic manifestation (AD diagnosis) providing novel information to the AD literature. Additionally, Aβ misfolding in blood plasma presents a unique marker of secondary structure changes of Aβ in blood plasma and beginning pathological changes associated to AD. Finally, the AD PRS is based upon the most recent GWA data^6^ and the investigation of an Aβ PRS is novel.

Several limitations of the study include the basis of the Aβ PRS, which was CSF measured Aβ and may therefore be not directly comparable to Aβ misfolding. Additionally, the nested case-control cohort study utilized for these analyses was only a small sample of the complete ESTHER cohort study with a much higher percentage of dementia cases than the entire cohort; however, similar associations between the AD PRS and dementia were evident in the entire cohort^46^. Another limitation includes the possibility of dementia misdiagnosis/underdiagnosis. The dementia diagnoses made in the ESTHER study were clinical diagnoses reported heterogeneously by numerous practitioners, and may be inferior to diagnostic standards that can be achieved in highly specialized academic settings. This is however the nature of community-based cohort studies, which portray common practice in such a setting. Also, the relatively small sample size and low number of AD and VD cases limited the power of the study and the generalizability of the results is limited to individuals of European descent.

## Conclusion

Alzheimer’s genetic risk, defined by an AD PRS and *APOE4*, was significantly associated with Aβ misfolding, an early blood marker of AD associated pathology, in a community-based cohort of older adults, albeit somewhat inconsistently. An association between an Aβ PRS and Aβ misfolding was not evident, however, the Aβ PRS was predictive of AD diagnosis within 14 years. Aβ misfolding was much more predictive of AD than any of the genetic risk markers, asserting itself as a viable AD risk marker. Further research should thoroughly evaluate and compare the potential of risk stratification by genetic risk markers and Aβ misfolding for more effective and cost-effective targeted measures of precision prevention and disease-modifying treatment.

## Supplementary information

Supplementary Figure 1, Supplementary Table 1, Supplementary Table 2
