# Supplementary material for: Genetic predisposition, Aβ misfolding in blood plasma, and Alzheimer’s disease
Source: Transl Psychiatry. 2021 May 1;11:261. doi: 10.1038/s41398-021-01380-0 (PMC8088439; doi:10.1038/s41398-021-01380-0)
Supplement: Supplementary file 1 — Supplementary Figure 1, Supplementary Table 1, Supplementary Table 2 [file 41398_2021_1380_MOESM1_ESM.docx]

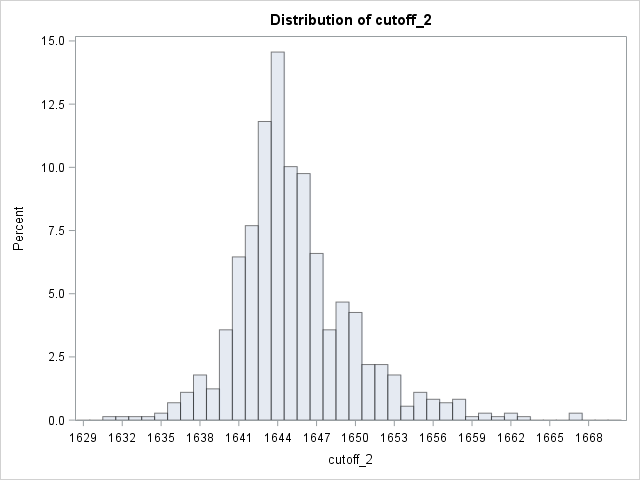

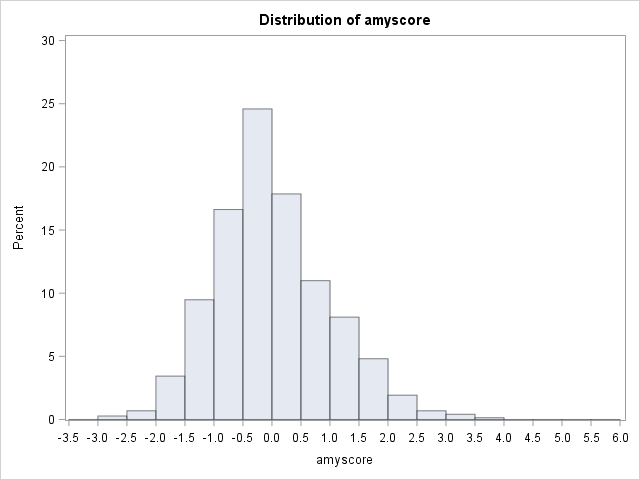

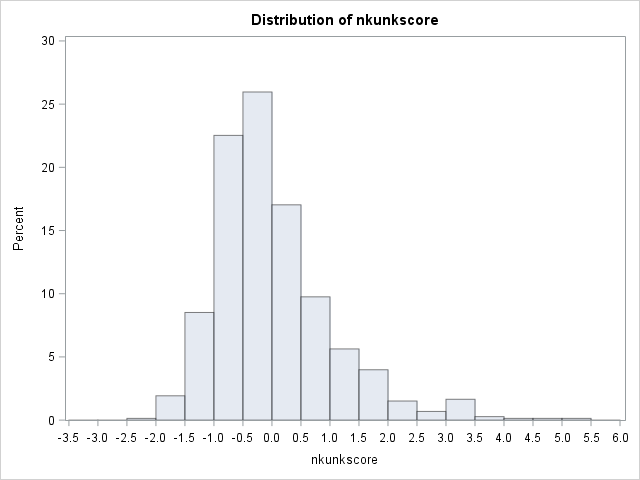


**A**

**B**

**C**

**Distribution of Aβ misfolding**

**Distribution of AD PRS**

**Distribution of Aβ PRS**

Aβ misfolding

Aβ PRS

AD PRS

**Supplementary Figure 1**: Plots of the distribution of the Aβ polygenic risk score (PRS), Alzheimer’s disease (AD) PRS, and Aβ misfolding. (A) Distribution of Aβ PRS. Participants with a PRS of 0.55 or above were considered Aβ PRS+. (B) Distribution of AD PRS. Participants with a PRS of 0.41 or above were considered AD PRS+. (C) Distribution of Aβ misfolding (cm^-^1). In agreement with a previously validated spectral threshold,^18^ participants with a cutoff of <1642 cm^-1^ were considered Aβ misfolding positive or to have high misfolding. Those participants at or above the validated spectral threshold were considered Aβ misfolding negative or to have low misfolding.

| **Supplementary** **Table 1:**  Logistic regression results: Association between Alzheimer’s and Aβ polygenic risk scores (quintiles) and Aβ misfolding | | | | | | | | | | |
| --- | --- | --- | --- | --- | --- | --- | --- | --- | --- | --- |
|  | All, n = 728 | | | | | Participants without dementia diagnosis, n = 581 | | | | |
|  | n, Aβ+ | n, Aβ- | OR (95% CI) | p-value | | n, Aβ+ | n, Aβ- | OR (95% CI) | p-value |  |
| Aβ PRS Q1 | 26 | 120 | Ref. |  | | 13 | 102 | Ref. |  |  |
| Aβ PRS Q2 | 24 | 122 | 0.89 (0.48-1.68) | .73 | | 12 | 105 | 0.86 (0.36-2.06) | .73 |  |
| Aβ PRS Q3 | 22 | 123 | 0.80 (0.42-1.51)  0.44 (0.22-0.92) | .48 | | 15 | 99 | 1.25 (0.54-2.90) | .60 |  |
| Aβ PRS Q4 | 13 | 133 | 0.44 (0.22-0.92) | .03 | | 10 | 117 | 0.65 (0.26-1.61) | .35 |  |
| Aβ PRS Q5 | 29 | 116 | 1.16 (0.63-2.13) | .63 | | 12 | 96 | 0.97 (0.40-2.35) | .95 |  |
|  |  |  |  |  | |  |  |  |  |  |
| AD PRS Q1 | 20 | 125 | Ref. |  | | 13 | 102 | Ref. |  |  |
| AD PRS Q2 | 22 | 125 | 1.16 (0.59-2.26) | .67 | | 14 | 109 | 1.06 (0.46-2.43) | .89 |  |
| AD PRS Q3 | 19 | 126 | 1.03 (0.52-2.05) | .94 | | 11 | 109 | 0.91 (0.38-2.20) | .84 |  |
| AD PRS Q4 | 23 | 122 | 1.31 (0.67-2.54) | .43 | | 11 | 103 | 0.87 (0.36-2.10) | .76 |  |
| AD PRS Q5 | 30 | 116 | 1.76 (0.93-3.32) | .08 | | 13 | 96 | 1.05 (0.45-2.49) | .91 |  |
|  |  |  |  |  | |  |  |  |  |  |
| Model covariates included age, sex, education  and 10 principal components.  Aβ+, Aβ misfolding positive  Aβ-, Aβ misfolding negative | | | | | *APOE4 +*, apolipoprotein E ≥1 ε4 allele  PRS, genetic risk score  Ref., reference  SD, standard deviation | | | | | |

| **Supplementary** **Table 2:**  Logistic regression results: Association between Alzheimer’s and Aβ polygenic risk scores (quintiles) and Alzheimer’s disease and vascular dementia diagnosis | | | | | | | | | | |
| --- | --- | --- | --- | --- | --- | --- | --- | --- | --- | --- |
|  | Alzheimer’s disease | | | | | Vascular dementia | | | | |
|  | n, AD | n, ND | OR (95% CI) | p-value | | n, VD | n, ND | OR (95% CI) | p-value |  |
| Aβ PRS Q1 | 12 | 115 | Ref. |  | | 9 | 115 | Ref. |  |  |
| Aβ PRS Q2 | 12 | 117 | 0.99 (0.42-2.35) | .98 | | 8 | 117 | 0.84 (0.30-2.36) | .74 |  |
| Aβ PRS Q3 | 9 | 114 | 0.83 (0.33-2.08) | .69 | | 13 | 114 | 1.64 (0.65-4.15) | .29 |  |
| Aβ PRS Q4 | 5 | 127 | 0.40 (0.13-1.18) | .10 | | 12 | 127 | 1.19 (0.47-3.05) | .71 |  |
| Aβ PRS Q5 | 21 | 108 | 2.03 (0.93-4.43) | .08 | | 12 | 108 | 1.41 (0.55-3.62) | .47 |  |
|  |  |  |  |  | |  |  |  |  |  |
| AD PRS Q1 | 9 | 115 | Ref. |  | | 9 | 115 | Ref. |  |  |
| AD PRS Q2 | 10 | 123 | 1.12 (0.43-2.92) | .81 | | 6 | 123 | 0.64 (0.21-1.91) | .42 |  |
| AD PRS Q3 | 6 | 120 | 0.69 (0.23-2.03) | .50 | | 14 | 120 | 1.35 (0.54-3.36)  35 () | .52 |  |
| AD PRS Q4 | 14 | 114 | 1.79 (0.73-4.36) | .20 | | 13 | 114 | 1.62 (0.64-4.08) | .31 |  |
| AD PRS Q5 | 20 | 109 | 2.52 (1.08-5.88) | .03 | | 12 | 109 | 1.25 (0.49-3.20) | .64 |  |
|  |  |  |  |  | |  |  |  |  |  |
| Model covariates included age, sex, education  and 10 principal components.  AD, Alzheimer’s disease  *APOE4 +*, apolipoprotein E ≥1 ε4 allele | | | | | ND, without dementia diagnosis (no dementia)  PRS, genetic risk score  Ref., reference  SD, standard deviation | | | | | |
